# Collaborating with a Youth Council to Improve Chronic Pain Resources

**DOI:** 10.1080/24740527.2023.2254358

**Published:** 2023-09-01

**Authors:** Kristy Wittmeier, Cara Brown, Francis Diaz, Heidi Pylypjuk, Gayle Restall, Polina Anang, Kerstin Gerhold

**Affiliations:** a Children’s Hospital Research Institute of Manitoba, Winnipeg, Manitoba, Canada; b Department of Pediatrics and Child Health, Rady Faculty of Health Sciences, University of Manitoba, Winnipeg, Manitoba, Canada; c Department of Occupational Therapy, College of Rehabilitation Sciences, Rady Faculty of Health Sciences, University of Manitoba, Winnipeg, Manitoba, Canada; d Department of Psychiatry, University of Manitoba, Winnipeg, Manitoba, Canada

**Keywords:** Chronic pain, youth, resources, engagement, mixed methods

## Abstract

**Background:**

There is a recognized need to involve people with lived experience of chronic pain when developing chronic pain resources.

**Aims:**

The aim of this study was to develop, implement, and evaluate a short-term youth council focused on eliciting youths’ recommendations for key features of chronic pain informational resources.

**Methods:**

In this mixed methods instrumental case study, demographic data were collected via Survey Monkey®. Select Patient-Reported Outcomes Measurement Information System® brief measures were used to provide context regarding pain impact within this group. Participants completed an initial interview, which informed youth council workshop delivery. Over two youth council workshops, participants reviewed select informational resources and identified key features of chronic pain resources. Participants evaluated their involvement experience during a second interview. Qualitative data were transcribed and analyzed using directed content analysis. Member-checking occurred during a third workshop, held virtually.

**Results:**

Seven youth self-identifying as girl/woman or demi-girl participated. The youth were satisfied with the youth council experience, highlighting the importance of meeting others, a relaxed environment, and participating in valuable work. A list of youth-identified key features for informational resources was created through the workshops, which includes considerations for audience groups, content, and presentation.

**Conclusion:**

Participants’ input into youth council development and meeting others with lived experience contributed to a safe and supportive involvement experience. Youth council involvement supported the development of preliminary recommendations for chronic pain informational resources.

## RÉSUMÉ

**Contexte:** Il est largement reconnu qu'il est essentiel d'impliquer les individus ayant une expérience personnelle de la douleur chronique dans le processus de création de ressources sur la douleur chronique.

**Objectifs:** Cette étude visait à établir, mettre en œuvre et évaluer un conseil consultatif composé de jeunes, à court terme, dans le but d'obtenir leurs recommandations concernant les caractéristiques essentielles des ressources d'information sur la douleur chronique.

**Méthodes:** Dans le cadre de cette étude de cas instrumentale utilisant une approche mixte, des données démographiques ont été collectées via Survey Monkey®. Des mesures brèves du Système d'information sur les mesures des résultats déclarés par les patients® ont été sélectionnées pour contextualiser l'impact de la douleur au sein de ce groupe. Les participants ont ensuite complété un entretien initial, qui a servi de base pour la mise en œuvre de l'atelier du conseil des jeunes. Au cours de deux ateliers du conseil des jeunes, les participants ont examiné des ressources d'information sélectionnées et ont déterminé quelles devaient être les principales caractéristiques des ressources en matière de douleur chronique. Les participants ont évalué leur expérience de participation au cours d'un deuxième entretien. Les données qualitatives ont été transcrites et analysées à l'aide d'une analyse de contenu dirigée. Une vérification par les membres a été effectuée lors d'un troisième atelier, qui s'est tenu virtuellement.

**Résultats:** Sept jeunes s'identifiant comme filles/femmes ou demi-filles ont participé à l’étude. Les jeunes se sont déclarés satisfaits de leur expérience au sein du conseil des jeunes, soulignant l'importance de rencontrer d'autres jeunes, d’un environnement détendu et de la participation à un travail utile. Une liste des caractéristiques clés identifiées par les jeunes pour les ressources d'information a été dressée au cours des ateliers. Celle-ci comprenait notamment des considérations relatives aux groupes cibles, au contenu et à la présentation.

**Conclusion:** La contribution des participants à l'élaboration du conseil des jeunes et la rencontre avec d'autres personnes ayant une expérience personnelle ont contribué à une expérience de participation sûre et positive. La participation du Conseil des jeunes a contribué à l'élaboration de recommandations préliminaires concernant les ressources d'information sur la douleur chronique.

## Introduction

There is growing global recognition of the importance of appropriately supporting children and adolescents who live with chronic pain.^1^ It is estimated that 20% to 25% of children or adolescents are affected by chronic pain at any given time^2^ and that 5% are affected to an extent that significantly limits daily functioning, including the ability to participate in school, physical activity, and other social activities.^3^ An interdisciplinary, multimodal approach based on the biopsychosocial model of care is currently recommended as the gold standard for pediatric chronic pain management.^4–6^ Access to this level of care, however, remains variable.^7–10^

A lack of resources about chronic pain, developed specifically for younger people, has compounded the issue of variable access to interdisciplinary pediatric chronic pain care in Canada.^11,12^ A series of reports, produced by the Canadian Pain Task Force between 2019 and 2021,^10,13,14^ highlighted the lack of accessible health services and long wait times for health services across age groups. Children and adolescents were noted to be an important and underrecognized group affected by chronic pain.^10^ The reports emphasized the need for improved information about chronic pain for health care providers and the public and the need to involve people with lived experience of chronic pain to fully understand the impact of chronic pain and to co-create solutions and strategies that will be relevant and impactful.^10,13^

The need for improved knowledge and awareness about chronic pain in children and adolescents was also highlighted within the Partnering for Pain study.^11^ This study used a modified James Lind Alliance approach to determine the top ten priorities for pediatric chronic pain in Canada.^11^ The James Lind Alliance is a nonprofit organization that has outlined methods for “priority-setting partnerships” that are designed to bring together people with lived experience, health care providers, researchers, and other interested parties to determine and prioritize “uncertainties” in a given area to direct future research.^15–17^ The Partnering for Pain study included researchers, health care providers, and children and adolescents with lived experience of chronic pain and their family members from across Canada. Using data gathered through a national survey and through a series of in-person workshops, this team generated a list of ten prioritized research questions to guide future pediatric chronic pain work.^11^ These research questions, or “uncertainties,” spanned the health, education, and government systems and collectively called for focused research to improve prevention, diagnosis, and management/treatment of chronic pain. Two of the ten priority questions generated through the Partnering for Pain study target improving knowledge and awareness of the validity of chronic pain in children and adolescents.^11^

With growing recognition of the gaps in chronic pain resources and services for children and adolescents, it is vital to include children and adolescents in co-creating solutions. Researchers and health care professionals must also learn from children and adolescents about how best to support their participation in co-creating solutions. The purpose of this study was to develop, implement, and evaluate a short-term youth council to elicit youths’ opinion on the beneficial features and gaps in a selection of existing chronic pain-related informational resources. In this article, we report on the development and evaluation of youth council workshops and present the key recommendations from this council as preliminary recommendations that can be used to inform future research and resource development.

## Materials and Methods

### Development of the Research Question

This study was informed by a local half-day research priority-setting event with youth who live with chronic pain and their family members. The event was supported by a grant designed to support the involvement of people with lived experience *prior to starting a study*. Funding allowed us to compensate those who attended the event and to purchase facilitation supplies (poster boards, markers, etc.) and refreshments. The event was conducted by members of the research team to learn about families’ priorities for research that could improve clinical services for chronic pain for children and adolescents at an emerging pain clinic within a tertiary care pediatric hospital. Families with a child who was seen at the emerging chronic pain clinic were invited to attend this event. Thirteen families took part. Demographic data were not collected from these families.

During the research priority-setting event, separate facilitated sessions were held for parents and children/adolescents. A large group discussion was held toward the end of the event to share perspectives and identify common topics between the parents and children/adolescents and key overarching topics. A summary of the session findings was shared with family members via e-mail afterward, with an opportunity to provide further input via e-mail or participation in a teleconference. Ultimately, three priority topics were identified: (1) the importance of the child’s or adolescent’s perspective on chronic pain, (2) the need for improved access to relevant resources, and (3) the value of peer-to-peer interaction. The present study was developed based on these priorities. Specifically, we wanted to hear from a group of adolescents about how to make more relevant resources, using a method of involvement that would also address the desire for more interaction with peers. Youth council workshops were selected as the method to facilitate youths’ participation in this work.^18,19^ This was to address the abovementioned family-identified priorities of including the youths’ perspectives and to provide an opportunity for peer-to-peer interaction.

### Study Design and Recruitment

A mixed methods instrumental case study design was used, because the instrumental case study is particularly useful “to gain a broader appreciation of an issue or phenomenon.”^20,21^ We used predominantly Western research methodologies. Families who attended the research priority-setting event were provided information about this project and the opportunity to participate. Study information was also included in a mail-out to families who had been referred for pediatric pain management at the same tertiary care center as families who attended the priority-setting event. In addition, recruitment posters were hung in the waiting area of the physician who was seeing children and adolescents with chronic pain (K.G.) and were posted on the clinic’s website. We aimed to recruit six to eight individuals, aged 14 to 18 years, with lived experience of chronic pain, aligning with common practice for group size in research^22^ and clinical practice for group sessions.^23^ The age range was expanded to 12 to 19 years during recruitment to reach the intended group size. Though the age range of our participants aligns with the World Health Organization’s definition of “adolescents,” from here forward we use the term “youth” in its colloquial sense in reference to the participants, to align terminology with the “youth council” used for this work. This study was approved by the Health Research Ethics Board at the University of Manitoba (H2018:417) and the Winnipeg Health Sciences Center Pediatric Impact Review Committee (RI2018:145). Participants 18 years of age and older provided written informed consent prior to participation. Youth <18 years provided assent and a parent provided written informed consent for the youth’s participation. An honorarium was provided to all participants for each interview and workshop that they took part in.

### Theoretical Frameworks

Development and evaluation of the youth council workshops were guided by a conceptual framework previously described and validated by G. Restall through research that engaged health care service users in mental health policy development.^24^ This framework provides a structure for exploring the social (normative, instrumental, substantive) and personal outcomes that can be expected to result from a participatory process, such as youth council workshops. Restall organized these outcomes using Bronfenbrenner’s ecological systems theory^25^ as microlevel (personal), mesolevel (instrumental, substantive), and macrolevel (normative) and provided questions to guide engagement development and evaluation that correspond to each of these factors.^24^ We used, and in some cases slightly adapted, the guiding questions to inform the youth council development and evaluation.

Additional guiding questions were included to support a trauma-informed approach to this work. These questions were drawn from recommendations for a trauma-informed intersectional approach for involving people with lived experience in health research.^26^ In their paper describing these concepts in relation to research, Shimmin and colleagues highlight the need to recognize that the lived experiences of people engaging in research may be intertwined with trauma.^26^ We included these additional questions in an effort to further promote a space in which youth could interact with the facilitators and other participants while feeling as safe and comfortable as possible. A matrix was developed from these two theoretical frameworks to inform the pre- and post-workshop interview guides and workshop development (Table 1; interviews described below). In addition to these guiding frameworks and concepts, a fundamental component of this study was that from inception to completion, youth were explicitly recognized as experts on their experience of chronic pain.

**Table 1.** Theory-informed matrix of guiding questions for development and evaluation of youth involvement.

Outcomes	Development questions^a^	Evaluation questions^a^
Microlevel (personal)	How do we (support) youth living with chronic pain through involvement mechanisms?	Did youth feel supported throughout the engagement process? Do they feel better equipped to manage pain?
	How do we manage personal risks for all participants?	Were the personal risks to all participants managed to the satisfaction of all involved?
	*How can we ensure that everyone involved (researchers and youth) feels safe, emotionally, physically, and psychologically? (T)*	*How did the research team work together to define and ensure emotional, physical, and psychological safety of all members of the research team?*
	*What are my personal values, experiences, interests, and beliefs about people living with (pediatric) chronic pain? (R)*	*How have personal assumptions changed? Why?*
Mesolevel (instrumental)	What is a communication strategy that will result in transparency of decision-making processes?	What communication occurred as a result of the involvement and decision-making process?
		To what extent did youth and researchers contribute to developing, implementing, and evaluating communication strategies?
	*What assumptions do you think underlie the representation of pediatric chronic pain? Who has framed it this way, and why? How has understanding changed over time? (R)*	*How may this project change assumptions of pediatric chronic pain? What (else) would you like to see change in the future?*
	*How can we ensure that everyone’s perspectives (including nonparticipants) are included? (I)*	*How were perspectives of others (not participating) considered or included?*
Macrolevel (substantive, normative)	What is the project goal?	Was the project goal achieved?
	How do we increase participation of youth in clinical/research advisory roles? *How do we address inequities and issues of social justice? (I)*	How many youth participated in involvement mechanisms? Are these youth interested in continued involvement? Are there fewer barriers to youth participation? *How was inequity or issues of social justice considered or addressed?*

Questions were used verbatim from the source where able, to align with the original framework; adapted as needed to reflect the context of this study.

^a^Items in non-italicized font are from Restall et al.^24^ and italicized items are from Shimmin et al.^26^

(R) = reflexivity, (I) = intersectionality, (T) = trauma-informed; overlap can occur.

### Data Collection

#### Demographic and Participant Health Related Data

Demographic data were collected via Survey Monkey®. Participants used codes instead of their names when completing the surveys to maintain confidentiality. All youth completed the Patient-Reported Outcomes Measurement Information System (PROMIS®) brief measures for anxiety, depression, fatigue, pain behavior, pain interference, and peer relationships to provide context regarding the impact of pain within this group.^27,28^

#### Interviews

Each youth was interviewed twice: once prior to participating in the youth council workshops and once after the second workshop. All interviews were completed by a single interviewer (F.D.) who was not involved in the workshops. The interviewer used a semistructured interview guide that was informed by the frameworks described above. Interviews were audio recorded with the participant’s permission. Questions in the first interview were designed to understand the youths’ perceptions about chronic pain and resources for pediatric chronic pain, to appreciate their expectations for involvement in the project, and to plan how to help youth feel supported during participation. These findings were used to help design the youth council workshops. The second interview focused on understanding the youths’ experience of participating in the workshops.

#### Workshop Field Notes

Field notes were taken by the facilitators during the workshops. Participants were invited to share written or verbal feedback at the end of each workshop. These data were also maintained for analysis.

In this article, we present the data pertaining to the youths’ perspectives on chronic pain-related resources, their list of key features for resources, and the development and evaluation of the youth council. Detailed information pertaining to the resource review is provided as Supplementary Material. The results of the youths’ perspectives on chronic pain and the specific interview questions used in that work have been published elsewhere.^29^

### Youth Council Workshops

The concept of the youth council as used in this study was informed by the rich descriptions of youth councils, advisory panels, and youth groups, provided by Coad et al.^18^ Bowen et al.^30^ and Brown et al.^19^ and by the broader patient and youth engagement literature.^31–34^ Coad et al.^18^ Bowen et al.^30^ and Brown et al.^19^ all described working with groups of youth for health system redesign, through various mechanisms that included youth council/advisor meetings or youth workshops. Though the youth councils/groups described by these authors were typically for a longer-term commitment (ranging from nine sessions to multiyear involvement), we opted for a short-term youth council given the focused nature of this study and the time-limited nature of the project and funding.

**Figure 1.**
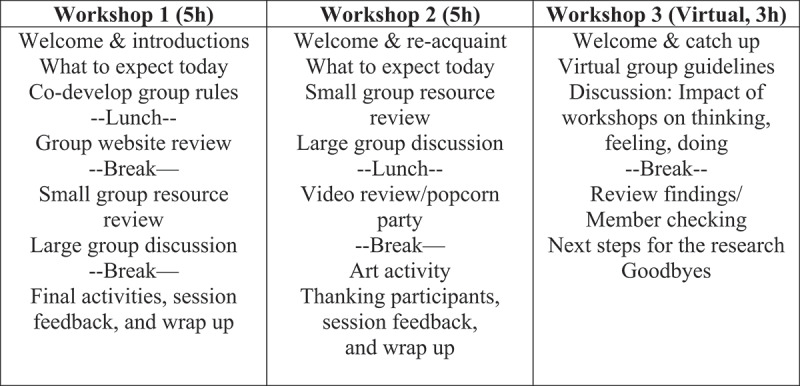
Outline of workshops.

Workshop agendas and activities were developed and facilitated by two research team members (C.B., K.W.), both of whom are rehabilitation clinician researchers with clinical experience with people who live with chronic pain (Figure 1). C.B. has clinical and research experience in facilitating group sessions and led the parent session and large group discussion portions of the research priority-setting event. The workshops were developed to account for participant needs, preferences, and expectations, as expressed in their initial interviews. The importance of group process was acknowledged in workshop development^35,36^; thus, group development activities were integrated throughout the workshop. Workshop activities were designed to be practical and engaging.^30^

All youth council workshops were held on a Saturday or Sunday from 11 a.m. to 4 p.m. local time. Weekends were selected so that participants would not have to miss school. Start times were chosen to accommodate families who were driving further distances for the sessions and with an appreciation that an early weekend morning may not be an appealing or viable option for youth. The sessions were held within a large open-space meeting room at the local pediatric research institute. The research institute is affiliated with and near the tertiary care center where pain services were provided. It was selected over a hospital space to reduce the potential impact of the medical environment on the youths’ participation experience.

The first two workshops were in-person, held 2 weeks apart (September 2019). During these initial workshops, participants reviewed select chronic pain-related resources (see Supplemental Material). A limited selection of materials was chosen by members of the study team for review during the workshops to inform the youths’ recommendations for future chronic pain resource development. Resources were selected with breadth of exposure in mind; that is, to include a variety of mediums, audiences, and level of detail in the resource. The resources included paper resources (e.g., workbooks, journals, posters), videos, and a local website. Sources included reputable pediatric health/pain associations from Children’s Healthcare Canada and Neuro Orthopedic Institute Group Australia and local or online retailers (Appendix 1). Resources also varied in the level of interactivity, in target age group, and in specificity to chronic pain (e.g., versus common co-occurring conditions such as anxiety). Variety in format, intended audience, and content was prioritized when selecting the material to elicit broad thinking related to resource development, resource sharing, and resource use in the clinical setting. Resources were not selected with the intention of recommending these for use in the clinical setting over others that were not reviewed. Participants were provided with scoring instruments specific for each type of tool (e.g., paper, video, website) to facilitate a more rigorous review and discussion (see Appendices 2 and 3 for more details).

The first workshop began with a review of the study aims. Participants then took part in an icebreaker to facilitate rapport building. For this, each youth was asked to choose one art card from a selection of cards distributed across a table. Once everyone had selected a card, each person was invited to introduce themselves, show the group the card they chose, and say something about why the card/image stood out to them. Next, ground rules for participation were set by the youths, with assistance from the facilitators. These were documented on a poster board and hung in the room for both in-person workshops.

The resource review started with a large group activity, during which all youth reviewed the chronic pain information on a local website. We started with a large group activity to encourage discussion and familiarity among participants. This was balanced by smaller group work later in the day, during which youth reviewed resources in groups of two or three. The day ended with a group check-in to hear what participants felt went well and what could be improved for the next workshop. Participants could share verbal feedback or write their feedback on a piece of paper for a facilitator to share with the group.

The second workshop began with the facilitators providing a summary of “what we heard” about chronic pain resources from the youth in the first workshop. The order of large versus small group review of resources was reversed for the second workshop, because youth had begun to establish familiarity by this time. The group activity for the second workshop was to review online (primarily video-based) resources together as a “popcorn party.” This was followed by an arts-based activity to mark the closure of the formal work of reviewing resources. The session was facilitated by an art therapist. Youth were provided the opportunity to draw an image that represented a significant time in their lives, a second image representing the present, and a third image of a time in the future. Youth had the option to share their art with the group and describe the images. Youth took their art home, and the art was not considered as data for the present study. Lastly, feedback was again sought from participants at the end of the workshop. Lunch was provided on both days, and frequent breaks were built in and adhered to.

A third and final workshop was held in April 2020, 7 months after the second workshop. The aims of the third workshop were to validate researcher-derived themes from the data, to discuss next steps, and to serve as a celebratory event. This workshop was held virtually using Zoom for Healthcare instead of in person due to COVID-19 pandemic restrictions.^37^ The researchers presented the preliminary results from the interview and workshop data analysis, and youth were invited to share their feedback on the accuracy of the findings and any further thoughts on their involvement experience.

### Data Analysis

Demographic data were analyzed using descriptive statistics and are presented as a high-level summary to preserve anonymity. PROMIS® questionnaires were analyzed using the HealthMeasures Scoring System (described elsewhere).^29,38^

Interviews recordings were transcribed verbatim by a professional transcriptionist and the anonymized transcripts were analyzed using directed content analysis methods.^39^ The analysis team included F.D. (interviewer), K.W. and C.B. (workshop facilitators), and G.R. Initial coding was completed by F.D. and K.W. after thorough reading and rereading of transcripts. The initial coding scheme was developed using key topics from the engagement framework matrix (Table 1), with additional codes and subcodes identified through line-by-line analysis of the interview transcripts. Codes were grouped into categories for comparison and interpretation. Facilitator notes taken during the group session were analyzed in a similar fashion. Preliminary findings were presented to the analysis team, and C.B. and G.R. provided additional insight. The coding frame was finalized and applied to the transcripts in NVivo 12 to facilitate reporting.^40^ Qualitative data were triangulated by comparing findings from the interviews and in-session data collection tools (described in Appendix 2) and facilitator notes. Of these data sources, data from the interviews and data collection tools were considered most strongly, because these data were directly from youth. Facilitator notes were used to provide additional context, clarification, or confirmation of findings. Feedback received from the participants during the third workshop was incorporated into the final analysis to define topics.

## Results

### Demographics

Seven youth aged 12 to 19 (median 15) years participated in the study; five lived within the province’s major urban center and two lived in smaller communities. Given the recruitment methods, all youth had been seen by the physician at the pediatric pain clinic. Youth reported having lived with chronic pain from 3 months to 5 years (median 3 years). Participants self-identified as girl/woman or demi-girl.^a^ Ethnic origins were self-reported as European or Canadian. None of the youth had previously participated in research. All seven youth attended the first two workshops. Five of the youth attended the third (virtual) workshop.

Results of PROMIS® questionnaires are presented in Table 2. Interpretations of these scores demonstrated severe impairment related to anxiety, depression, and fatigue; moderate impairment related to pain interference; mild impairment in pain behaviors; and fair peer relationships.

**Table 2.** PROMIS scores and interpretation.

PROMIS scale	Median (IQR)	PROMIS T-score interpretation^a^ (range)
Anxiety	66.5 (12.4)	Severe (mild–severe)
Depression	70.1 (11.6)	Severe (moderate–severe)
Fatigue	67.2 (7.4)	Severe (moderate–severe)
Pain behavior	53.7 (2.2)	Mild (within normal limits–moderate)
Pain interference	64.3 (2.0)	Moderate (moderate–severe)
Peer relationships	36.8 (12.1)	Fair (poor–good)

^a^The PROMIS® scoring guide cautions that these are general labels to assist with interpretation, and thresholds may be different for various conditions. IQR: Interquartile range

### Youth Council Workshop Development

Each of the seven participants completed both a pre- and post-workshop interview. The two major topics derived from the interview data relating to preparing for research participation were *motivation to participate* and *how to support my participation*.

#### Topic: Motivation to Participate

Most youth indicated that helping others who experience chronic pain was a prime motivator for participating in this research. They discussed how creating better awareness about chronic pain and having more information readily available could help more youth obtain a diagnosis faster, understand chronic pain better, and feel less alone.

I’d love to help out with the youth that are going through the same thing as me. And anything I can do to make it easier or better to help their experience or help them manage their pain and actually have a life with their pain … it would be great to help out. (P4)

Over half of the youth identified that meeting and learning from others who live with chronic pain was important to them. They expressed how difficult it was to explain their situation and that it was rare to feel understood by others. They also expressed an interest in learning how other young people with chronic pain dealt with day-to-day issues. Most had not met another youth who experienced chronic pain. Several youth indicated that they hoped they would find new resources that they could use and learn from through this research.

When they diagnosed me with whatever … there wasn’t a lot of people that I knew that were involved in what I had. And so, I was curious as to see people with similar effects that I have. So, I wanted to, to see other people that could help me, or I could help them understand what they’re going through, or help me [with] what I’m going through. (P7)

#### Topic: How to Support My Participation

Most of the youth expressed excitement about the upcoming research participation and had few concerns. When prompted to share even minor concerns, the most common response was that of shyness or that it might be difficult to talk or share their experiences in a group setting. Other comments related to not knowing exactly what to expect, the potential of not having answers to questions that are asked, and hoping that it would not be “*just like work, work, work*” (P2). Suggestions to make participation more comfortable included building group rapport or a level of comfort and familiarity between members and providing a relaxed space and process. Practical suggestions were provided and subsequently used by the facilitators, such as including icebreakers and having frequent breaks. Regarding physical space and process, as one participant put it, “*If it couldn’t feel like as medical as possible, that would be great*” (P3). Overall, youth expressed motivation to participate, even in the presence of concerns.

Um. Speaking and, um, talking about it is really hard as you can probably tell. … But I think if you don’t speak up about it, nothing’s ever going to change, so it’s worth getting worried about. (P7)

### Youth Council Workshop Evaluation

The data used to understand the participation experience included the within-workshop feedback (field notes and anonymous feedback) and the post-workshop interviews. Findings centered around two main topics: *the experience of the workshops* and *the value of the work.*

#### Topic: The Experience of the Workshops

I think the experience is the best part. Being with other youth that have chronic pain is crazy when you think about it but also really comforting. It gives you a chance to be like, hey, I’m not alone. There’s other people like me. … I think that was probably the biggest benefit. And the other one was just helping with the resources and website and stuff. (P1)

When asked to talk about their experience in this project, almost every participant began by reflecting on how much they enjoyed meeting other youth who have lived experience of chronic pain. Youth spoke about not needing to explain their situation in detail for others to understand and that it was easy to relate to one another. Participants commented on respectful communication within the group, that they felt they were with people they could trust, and that it was reassuring to hear from others who had experiences like their own.

**Figure 2.**
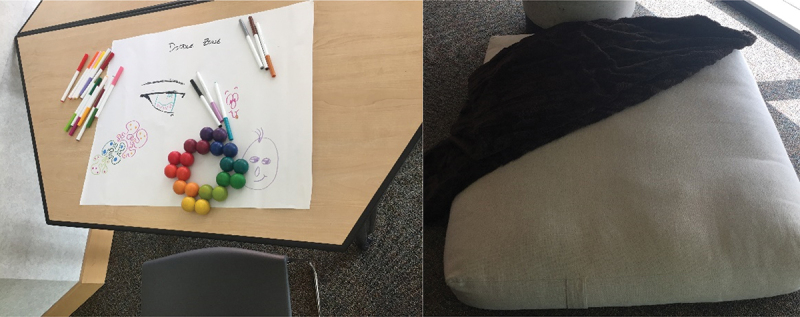
Photos of the workshop doodle station and flop zone.

All participants affirmed that they felt safe and supported. Some youth described developing a better understanding of chronic pain during the workshops. Some indicated that they were introduced to resources that they had not previously seen and had subsequently accessed these resources for personal use. The youth felt that their voices were heard, providing examples of how the workshop facilitators took notes throughout the session, responded to their suggestions, and discussed future plans. Several details in the physical space (Figure 2) and processes within the workshop were highlighted as contributing to feelings of comfort and safety.

And we were all comfortable. … They told us that we could leave and come back. That we can have food, water. They had this … cushion on the ground if we had to go and sit on the ground and just be alone for a little bit. They had a doodle station. So, they really prepared it for people with chronic pain and anxiety. So, we would be comfortable, and it’d be – they made it adaptable to us which was great. (P4)

When prompted by the interviewer for “downsides” of involvement, participants cited the long drive for those from out of town, as well as the need to rearrange individual/family schedules to support workshop attendance. This feedback was provided before youth experienced the use of virtual meeting technology for the third workshop. Though specific solutions to these issues were not identified, general ideas for workshop improvement included shorter and more frequent sessions, having more sessions, having the sessions closer together, and providing more comfortable chairs.

#### Topic: The Value of the Work

Most participants indicated that they felt this work could make an impact and help others, aligning with what was identified as a primary motivator for participation in this study. They recognized their lived experience as expertise that could improve resources and care for other youth with chronic pain. Several participants described a dual benefit of participatory research, in that it can benefit others and empower those who participate.

Not only does it help the future of other kids, but it also is something special for them, too, to be able to participate. Have their voices be heard. To meet other people so you’re not alone. It’s very … very special. (P4)

When asked about the role of research, participants indicated that research helped *keep things up to date* and *moving forward* and helped youth *to feel noticed*. In the post-workshop interviews, youth revisited the conceptualization of *knowledge as hope* that they discussed in the initial interviews, as reported in a previous manuscript.^29^ The feelings of hope persisted, and even strengthened for some.

Q: Uh huh. Um. Anything else that you got out of the project personally?

A: Um. I got hope. Uh. Hope that, you know, there’s going to be better resources for other kids. … Like who have chronic pain [and] … don’t have any like support systems out there or … people that they know that have chronic pain.

Q: And how important is hope?

A: Really important. It’s like top on the list of important things. … I don’t know how to describe it. It just the importance in like you don’t give up kind of thing. (P6)

### Perspectives on Chronic Pain Resources

Within the workshops and both interviews, participant discussion about resources revolved around two main topics: *resource gaps* and *language matters.*

#### Topic: Resource Gaps

Discussions about resource gaps mainly centered around a lack of youth-directed resources, lack of available resources in primary care settings, and a lack of resources to help youth explain their pain to others. In their initial interviews, the youth who discussed looking for information online around the time of their diagnosis mentioned that they mostly found information about chronic pain in adults, written for an adult audience. Accordingly, the resources used language that was often at a high level and lacked clarity, and resources were not always relevant for youth.

I tried, um, after learned about the word chronic pain, I was searching it. And I did find some good stuff, but it was mostly in, like, for adults. … Nothing with like kids or teens. (P1)

Youth indicated that it would be ideal to have resources available in primary care settings such as medical clinics and health facilities, rather than solely in pain clinics. Participants wanted family doctors or primary health care providers to share chronic pain resources with them and their family, indicating that having resources in a variety of health settings would help raise awareness about chronic pain and help youth feel less isolated.

Resources to help youth talk to teachers or to direct teachers to information about chronic pain were highlighted as especially needed. Most participants discussed having had experiences with teachers who did not believe that they had pain or that their pain was as severe as they reported it to be. Throughout the workshops, the youth participants identified many important features of youth-focused chronic pain resources. These were summarized and categorized by audience groups, content, and presentation and are presented in Table 3.

**Table 3.** Key features of youth-focused resources as identified by the youth council.

Audience groups	Children Teens and young adults Teachers Health care providers Public (including family and friends)
Content	Causes of chronic pain Variation in how pain presents Association between pain and mental health How to talk about chronic pain with others Identifying and managing pain triggers Treatment options Coping techniques (When able) how to fix the pain versus cope with the pain How to live with chronic pain in different phases of life, including the transition period between phases Preparing for postsecondary education/work
Presentation	Use language carefully and with sensitivity Write content specifically for a youth audience Avoid slang and jargon Include content and contributions from youth or have entire resources created by youth Validate the pain experience of youth Use visuals and a variety of formats (online, printed; text-based, video, etc.) Present options for pain management; make it clear that different things will work for different people Include actionable items where possible Be clear and honest when answers are not known

#### Topic: Language Matters

The importance of language and phrasing was discussed within the interviews and during all three workshops. Youth wanted clear and age-appropriate language but did not want resources to attempt to replicate the way a young person may speak to a friend. The use of slang in a professional resource was seen to diminish the resource’s credibility and appeal. Youth were also very attuned to wording that was seen to invalidate their experience; this was discussed at length. One participant stated the following about a sentence that had been used on the provincial website.

It was in … the diagnosis paragraph saying that … when you’re being diagnosed, um, you’re probably worried. This feeling is normal. That we didn’t like “this feeling is normal.” … And we felt like it was just dismissing our feelings and just saying like it doesn’t matter … we don’t care. (P2)

In the post-workshop interviews, the statement “this feeling is normal” was referenced by several participants. Some discussed how the initial wording was invalidating, emphasizing the importance of language in resources. In addition, youth used this example to demonstrate how they were able to make change through participating in research, by highlighting the issues with this statement and recommending that the wording be changed and that the changes were implemented.

## Discussion

This study was designed to respond to family-identified priorities for chronic pain research, by engaging with youth who have lived experience of chronic pain using a youth council format. The development, format, and evaluation of the youth council workshops have been described in detail for transparency to facilitate adaptation and use by others who are interested in engaging youth in clinical or research initiatives. The output of the workshops, specifically the recommendations generated by the youth in this study, can be used and built on to inform the development of chronic pain resources for youth, with specific attention to audience, content, and presentation. Content recommendations can also be used to assess whether a clinic or pain service has resources to address youth-identified areas of interest, making youth-created or youth-informed resources available when possible.

The youth council format responded to the priority of “more peer-to-peer interaction” identified by families in the local priority-setting workshop that led to this study. Within the follow-up interviews, the experience of meeting other youth with chronic pain was generally the first and most frequently cited benefit of participating. Young people who live with chronic pain have described experiences of peer isolation and pain invalidation.^41,42^ Participants in our study expressed feeling understood, validated, and being able to build rapport over the course of two half-day sessions. These findings align with those of Hall and colleagues,^43^ who reported on a 1-day migraine camp for parents and teens affected by chronic migraine.^43^ Social interaction was highly valued within these camps, with parents and teens both noting that the most important aspect was realizing that “they are not alone” in their experiences.^43^ However, it must be noted that a reflective approach must be used to promote the development of trust and safety in the group to obtain this result. In this project, the youth spoke highly of their experience in the youth council and affirmed feeling safe and supported. We attribute this, in part, to the approach taken to designing the workshops, including being informed by the youth themselves through their input during the initial interviews. In the post-workshop interviews, youth specifically identified workshop features such as the snacks and flop zone, which were intentionally included to support participants to feel safe and comfortable. Other strategies used included frequent breaks and attempts to balance more focused work such as the resource review with less structured activities such as the group discussion. We ensured that the sessions were facilitated by a team member who was experienced in group process and facilitation and familiar with a trauma-informed approach.^26^

Youth involved in this study recognized their agency and were eager to share their experience to improve resources and services for others. Increasingly, it is recognized that research, clinical approaches, and informational resources need to be available for and informed by children, youth, and young adults who live with chronic pain.^11,41,44^ Involvement can be viewed as important from both a rights-based and empirical perspective.^45^ Ozer et al. described the rights-based perspectives of involvement as providing a voice for youth in the development of services that they will use, flattening power hierarchies, and supporting diversity in inclusion mechanisms.^45^ The empirical perspective speaks to how youth involvement may improve the design, content, and effectiveness of programs accessed by young people and which programs or areas may benefit most from including the youth voice.^43^ In other words, youth have a right to participate in this work. Involvement that avoids tokenism and is a positive personal experience can result in substantive and instrumental outcomes^24^ of better and more legitimate resources. Youth and health care providers may be more likely to access and use the resources or find them more acceptable if they are aware that youth were involved in their development.

This study identified that important content for chronic pain resources includes causes of chronic pain, variation in presentation, coping techniques, treatment approaches, and the association with mental health. Of note, youth also identified the importance of content specific to different life transitions and phases (e.g., adolescence to adulthood). Some of the younger participants discussed the benefit of hearing from older participants within the group, appreciating their perspectives on topics such as postsecondary education and work. The older adolescent to young adult period is an important life stage that can be interrupted by the experience of chronic pain.^41,42^ In a review by Rosenbloom et al., the authors advocated for a developmental approach to pediatric chronic pain research tailored to support the achievement of meaningful milestones that tend to occur during this phase of life, such education, work, independence, and relationships.^42^

Since this study was conducted, a Canadian initiative, Solutions for Kids in Pain (SKIP; kidsinpain.ca) has been launched with the support of the Networks of Centers of Excellence. SKIP has a mandate to improve knowledge translation related to pediatric pain, both acute and chronic. This represents a major opportunity for the field, where gaps identified here and elsewhere can be brought forward to be addressed. SKIP is also a Patients Included Organization,^46^ which creates opportunities for youth and family members to be involved in resource development. As of the writing of this article, SKIP has adapted and published at least one resource that addresses both an audience and content topic identified through this study. The *Guide to Chronic Pain in Students*^47^ is a resource that includes information for teachers about chronic pain, as well as a template for a personalized “in-class pain plan” for students. This has the potential to be further adapted to include tips and prompts for youth who are looking for different ways to talk about chronic pain with other people, such as friends, family members, or employers.

As we have previously noted,^29^ limitations of our work include a small sample size with limited gender, racial, and ethnic diversity. Pain experiences and outcomes are documented to vary with gender, socioeconomic status, age, and race, which are intertwined with bias in health care that influences service accessibility and delivery.^48,49^ Our findings should be built upon with additional research with larger and more diverse groups of youth. Youth were also recruited from a single province with a limited chronic pain service.^9^ This single-province approach made it possible initially to hold in-person sessions; however, it also presents a bias in our findings toward the experiences of youth within this province. With the onset of COVID-19, the study methods were adapted to hold the last workshop virtually. Since that time, virtual engagement has become commonplace, which opens opportunities for engagement across a wide geography in future research.

Overall, this study illuminates the importance of creating and maintaining spaces for people with lived experiences to inform clinical resources and care. The youth involved in this study were motivated to share their expertise to help others and were eager to work alongside others with similar experiences. Their recommendations can be shared widely, built upon, and used to create resources that are more appealing, relevant, credible, and useful for young people with chronic pain. As stated by the young adult who reviewed this article prior to publication, “We want to be helped by people who understand.” Collaborative engagement between academic/clinical researchers and youth with chronic pain and the creation of more avenues to amplify the voice of young people will lead to learning, progress, and improved health care.

## Supplementary Material

Supplemental Material

## Note

[a]. “A gender identity term for someone who was assigned female at birth but does not fully identify with being a woman, socially or mentally” (see: https://web.uri.edu/gender-sexuality/resources/lgbtqa-glossary/trans-101-glossary/).^50^
