# Supplementary material for: Collaborating with a Youth Council to Improve Chronic Pain Resources
Source: Can J Pain. 2023 Sep 1;7(1):2254358. doi: 10.1080/24740527.2023.2254358 (PMC10614709; doi:10.1080/24740527.2023.2254358)
Supplement: Supplemental Material [file UCJP_A_2254358_SM5563.docx]

**Supplementary Material**

**Appendix 1. Resource list, source/author, and intended audience**

| **Resource and Source/Author** | **Intended Audience**  **(using language of resource creators, where specified or provided)** |
| --- | --- |
| Clinic Website (Chronic Pain section)  Everychildeverytime.ca | Families, general public |
| Ebook  One family’s story: Learning to live with chronic pain  echokt.ca  Resource by EchoKT, Stollery Children’s Hospital Foundation, Women and Children’s Health Research Institute | Parents of children who live with chronic pain |
| The Chronic Pain and Illness Workbook for Teens: CBT and Mindfulness-Based Practices to Turn the Volume Down on Pain  by Rachel Zoffness MS PhD | Teens |
| Neuro Orthopaedic Institute (NOI) Resources  Protectometer (book)  NOI Group Posters  Explain Pain textbook  https://www.noigroup.com  Resources by David S. Butler & G. Lorimer Moseley | People experiencing pain  Clinicians and patients  Clinicians, people in pain and their families |
| Video: Youth Voices  Part 1: Hiding the Struggle  Youtube.com  by Dr. Roberta Woodgate | Youth, families, teacher, healthcare providers, general public |
| Painbytes: School video  <https://aci.health.nsw.gov.au/chronic-pain/painbytes>  Agency for Clinical Innovation | Youth |

**Appendix 2: Evaluation of Chronic Pain-Related Resources**

Data Collection

Resource evaluation was gathered during youth council workshops. In addition to data collection tools described below, field notes were taken by workshop facilitators during the workshops. The Patient Education Materials Assessment Tool - Print (PEMAT-P) was used for review of print resources (books, posters),^1,2^, and the PEMAT-AV for audiovisual (AV) material (videos).^3^ The PEMAT is designed to score the *understandability* and *actionability* of education materials, which are each scored using a set of items to which users respond yes (=1), no (=0) or not applicable (N/A). A percentage is calculated and used for reporting purposes. Understandability is defined as the degree to which people can understand main concepts and messages of the resource, and actionability refers to the ability for people to identify steps or actions to take based on the resource.^1,4^ Both of these definitions are meant to apply to diverse groups of people across the spectrum of health literacy levels. A threshold of 70% has been proposed, such that scores 70% and higher indicate that material is understandable or actionable, however further empirical testing is required to validate this threshold.^1^ As recommended by the PEMAT development team,^1,4^ we developed a study-specific survey to supplement the PEMAT with additional questions to understand resource usability. Survey questions were adapted from a study evaluating usability for an e-book on chronic pain,^5^ and included: i) this resource is good for: (check all that apply: [me, other youth with pain, parents, teachers, doctors, my friends, other family members, other]), ii) this resource is relevant to me as a youth with chronic pain, iii) this resource is useful, iv) this resource is a good length, v) I like the way it looks. A 7-point emoji Likert scale was developed and used for questions ii-v, ranging from 1 (strongly disagree) to 7 (strongly agree), with space for open text responses. The local website was evaluated through group review and guided discussion, with prompts taken from the Michigan Consumer Health Website Evaluation Checklist.^6^ Videos were also reviewed as a large group. Remaining resources were initially reviewed by small groups of two to three youths. To maximize participants’ exposure to a variety of resources during the allotted time, everyone did not review and score each resource, however small groups presented the resource and their reviews back to the larger group for discussion.

Data Analysis

The PEMAT Auto-Scoring Form was used to derive understandability and actionability scores (%) for the resources that were reviewed using the PEMAT.^4^ To derive the percentage scores, item scores for understandability and actionability were summed, then divided by the number of response items. Values were then multiplied by 100 to obtain a percentage score.^4^ Median percentage scores were calculated for both PEMAT variables. Median Likert scale responses were calculated for reporting purposes.

Results and Discussion

Quantitative results of the within-workshop resource review are provided in **Appendix 3**. The resources that received the highest overall ranking via the combined Likert scales were a set of posters from the NOI that explain various aspects of chronic pain and rehabilitation (titled: Thought Viruses, Graded Activity, Road to Recovery, Take Control) and a narrated video about anxiety (Hiding the Struggle). These resources received median scores of 6 on the 7-point usability Likert scale and scored highest for understandability using the PEMAT. Youth described the NOI posters as *“helpful”,* providing *“good information”, “eye catching”, “accurate”,* and *“understandable”.* They indicated that they could identify with the information and liked the advice to speak about specific topics with their doctor. The video about anxiety was described as “*engaging”*, and “*good for a variety of audiences”*, including youth who are starting to understand their own diagnosis. It was noted that while the video did not focus on chronic pain, the experience of anxiety was one that some participants could strongly identify with. Youth found the visuals and special effects in the video impactful, and effective. The median actionability score for the posters was 75.5%, while actionability for the anxiety video was 0%.

The Chronic Pain & Illness Workbook for Teens, and The Explain Pain Handbook: Protectometer, scored highest for actionability (100%), with median understandability scores of 74% and 50%, respectively. The Workbook received high scores on usefulness questions, with median Likert scale scores ranging from 5 to 6. Youth appreciated the tools and activities and perceived the information to be accurate. The Explain Pain Handbook: Protectometer was rated to be the least useful by youth in this study, with median Likert scale scores ranging from 2 to 4. While the youthful look of the resource was appreciated, it was perceived as “*a little overwhelming”*, and less relevant for a youth audience, which of note, was not the main target audience of this resource.

The goal of this study was not to perform a comprehensive review of all pain resources, or to focus solely on pediatric pain resources. Rather our team decided to provide youth with a variety of resources to review, to promote discussion and identification of what they liked or did not like within the resources. The resources that had the highest Likert scale ratings in this study were not only deemed relevant to a young person, but also had visual appeal and clarity/understandability as common features. In fact, the resources that were ranked highest via the Likert scales (poster set, video) also received the highest scores for understandability (median PEMAT 90% for both). In contrast, actionability scores for these resources diverged considerably (poster set: actionability 75.5%; video: actionability 0%). These findings highlight that different resources can meet different needs, and that not all resources need to be actionable. While brief, clear, and actionable information made the posters resonate with youth, the video about anxiety presented information in a way that incorporated dance and story, and reflected young people’s experiences with anxiety in a relatable way.

The goal of this study was not to perform a comprehensive review of all pain resources, or to focus solely on pediatric pain resources. Rather our team decided to provide youth with a variety of resources to review, to promote discussion and identification of what they liked or did not like within the resources. From this, the participants were able to articulate what stood out to them as key features of chronic pain resources.

During the resource review, youth highly rated resources that had the combined features of being deemed relevant to a young person, having visual appeal, and high ratings for understandability. The preferred resources varied in their level of actionability, defined as ability for people to identify steps or actions to take based on the resource.^4^ This highlighted to our team that different resources can meet different needs, and that not all resources need to be actionable. Youth had a similar level of appreciation for a set of posters with brief, clear, and actionable information, and a video that shared young peoples’ experiences with anxiety through dance and story.^7^

A limitation of the resource review was that each youth did not have the chance to provide an in-depth review of every resource, as we chose to focus on introducing a variety of resources to facilitate dialogue and ensure time for peer-to-peer interaction.

**References for Appendix 2**

1. Shoemaker SJ, Wolf MS, Brach C. Development of the Patient Education Materials Assessment Tool (PEMAT): a new measure of understandability and actionability for print and audiovisual patient information. *Patient Educ Couns*. Sep 2014;96(3):395-403. doi:10.1016/j.pec.2014.05.027

2. Agency for Healthcare Research and Quality. PEMAT for Printable Materials (PEMAT-P). Accessed August 11 2020, <https://www.ahrq.gov/ncepcr/tools/self-mgmt/pemat-p.html>

3. Agency for Healthcare Research and Quality. PEMAT Tool for Audiovisual Materials (PEMAT-A/V). Accessed August 11 2020, <https://www.ahrq.gov/ncepcr/tools/self-mgmt/pemat-av.html>

4. Shoemaker S, Wolf M, Brach C. Patient Education Materials Assessment Tool and User's Guide. Rockville, MD: Agency for Healthcare Research and Quality; 2013.

5. Reid K, Hartling L, Ali S, Le A, Norris A, Scott SD. Development and Usability Evaluation of an Art and Narrative-Based Knowledge Translation Tool for Parents With a Child With Pediatric Chronic Pain: Multi-Method Study. *J Med Internet Res*. 12 2017;19(12):e412. doi:10.2196/jmir.8877

6. Anderson P. *Consumer health website evaluation checklist*. 2001. <http://www-personal.umich.edu/~pfa/pro/courses/EvalPtEd.pdf>

7. Woodgate RL, Tennent P, Legras N. Understanding Youth's Lived Experience of Anxiety through Metaphors: A Qualitative, Arts-Based Study. *Int J Environ Res Public Health*. Apr 19 2021;18(8)doi:10.3390/ijerph18084315

**Appendix 3: Rating and relevance scores for the resources reviewed by youth council participants.**

| **Scale** | **Provincial Website**  **(n=7)** | **E-book: One family’s story (n=4)** | **The Chronic Pain and Illness Workbook for Teens (n=3)** | **Explain Pain Textbook**  **(n=4)** | **Protect-ometer**  **(n=5)** | **NOI poster set**  **(n=4)** | **Hiding the struggle, video^b^ (n=7)** | **Pain Bytes School, video^b^**  **(n=7)** |
| --- | --- | --- | --- | --- | --- | --- | --- | --- |
| **Question (Likert scale, median score)^a^** | | | | | | | | |
| **This resource was relevant to me as a youth** | 4 | 5.5 | 5.5 | 4.8 | 2 | 6 | 6** | 3** |
| **This resource is useful** | 5 | 4.5 | 5 | 6 | 2 | 6 | N/A | N/A |
| **This resource is a good length** | 5 | 5.5 | 5 | 5.3 | 4 | 6 | N/A | N/A |
| **I like the way it looks** | 5 | 5 | 6 | 5.5 | 3 | 6 | N/A | N/A |
| **PEMAT (%)** | | | | | | | | |
| **Understand-ability**  **(PEMAT, %)** | N/A | 87% | 74% | 77.5% | 50% | 90% | 90% | 87% |
| **Actionability (PEMAT, %)** | N/A | 57% | 100% | 73.5% | 100% | 75.5% | 0% | 83% |
| **This resource is relevant for:^c^** | | | | | | | | |
| **“Me”** | ++ | ++ | ++ | ++ |  | ++ | ++ | ++ |
| **Other youth with pain** | ++ | ++ | ++ | ++ |  | ++ | ++ | ++ |
| **Parents** | ++ | ++ | + | ++ | + | ++ | ++ | ++ |
| **Teachers** | ++ |  |  | ++ |  | + | ++ | ++ |
| **Doctors** |  | + |  | ++ |  | ++ | ++ |  |
| **My friends** | ++ | + |  | + |  | + | ++ |  |
| **Other family members** | ++ | ++ |  | ++ |  | + | ++ |  |
| **Others (fill in blank)** |  |  |  |  |  |  |  | Other students |

^a^Likert scale: 1 *(strongly disagree)* to 7 *(strongly agree)*

^b^Only an overall score was provided by the youth for these videos, as they wanted more time for discussion of the videos. The relevant audience was decided by consensus through group discussion.

^c^Blank=no youth selected this audience, +=<50% of youth reviewers selected this audience, ++≥50% of youth reviewers selected this audience

NOI: Neuro Orthopaedic Institute

See Appendix 1 for sources and target audiences of resources
